# A Medical Causerie

**Published:** 1916-01-15

**Authors:** 


### A MEDICAL CAUSERIE.

Deputations from the Central Medical War Committee for England and Wales, and from the Scottish Emergency Committee, have had an official interview with Lord Derby, and it has been agreed that these two committees shall be recognised as the responsible authorities for organising the medical profession relative to the war. No results of the canvass to date have been published, but it is certain that many more practitioners will be required if the new Armies are to receive an adequate number of medical officers. The authorities are willing, so far, to leave the matter in the hands of the profession, but the numbers required will have to be forthcoming or the need for other methods will certainly be recognised. This necessarily means a demand for considerable activity on the part of the Medical War Committees already established throughout the country, and these are being urged to canvass personally all practitioners of military age in their corresponding districts. For men who feel unable to accept commissions at once—and more of these are urgently required—there is to be arranged a series of groups based upon a classification which places in the forefront junior and assistant medical officers, and arranges in series others according to the readiness or otherwise with which their work can be undertaken by colleagues. In short, the method adopted in Lord Derby's general recruiting scheme has been followed, and in this fashion all practitioners of military age will be made to feel the pressure. The plain fact is that the doctors must be obtained in one way or another, and at present the demands of the War Office are very far from satisfied.

In the Marylebone district it is claimed that out of some 1,100 practitioners nearly 300 already hold military commissions. Such an announcement has an impressive quality, but then it must be remembered that these figures include the holders of *à la suite* commissions, who, physicians and surgeons in the general hospitals, are also carrying on the work at the local military hospitals. The responsibilities of these last-mentioned positions are not slight, but they can for the most part be reconciled with the continuation of a private consulting practice, and they carry pay as well as fame. The men who suffer are the younger men who, with modest remuneration and rank, are sent to hospitals so remote from the area of their private work that all chance of consultation fees is denied them. The new canvass in Marylebone is to be conducted, it appears, by senior hospital men, many of whom have quite distinguished names. Whether these gentlemen are the persons the best suited to appreciate the difficulties of junior men engaged in general practice may be doubted, and some of the interviews proposed may not be without piquancy. Where precept cannot be supported by example it is dangerously open to a sharp retort, and the whole position would have been much easier had the proposal to institute a professional fund been adopted. By this arrangement there would have been at least the appearance of equality of sacrifice, and the corporate service of the profession to the community in the present national emergency would have been conspicuous.

When the time comes for an inquiry into the working of the medical activities of the National Insurance Act there will be some interesting revelations. There is no doubt that discontent with its provisions is growing both among the insured themselves and also among the panel doctors. It used to be said that under the old club system where the doctors supplied medicine to the patients there was a temptation to cheap prescribing and to the use of inferior remedies. The new era was to get rid of all this, and was to ensure everything of the finest and most superior quality. But nowadays, and especially with drugs rising in price, the unhappy doctor has constantly to remember that unless he keeps his prescribing expenses below a certain level he is liable to be challenged by the local Insurance Committtee. Even if he succeeds in satisfying the appointed tribunal he may well waste a number of hours in doing so, and should he fail he finds himself surcharged for extravagant prescribing. The effect of such a state of matters is obvious, for with the best will in the world no man can conduct his professional activities at the highest standard when he has to realise that a committee armed with a club is waiting for him round the corner. Practitioners may well feel and speak bitterly about such an arrangement, and it might be interesting for patients to be informed of the position and to be introduced to a certain insurance Pharmacopœia as encouraged in at least some districts and marked "for private circulation only." There are many things which are cheap and which merit also another and less complimentary adjective. Among these cheap medicines may certainly be numbered.

A students' journal connected with one of the London hospitals, in welcoming the newcomers at the beginning of the session, boasted of the traditions of the institution and reminded all and sundry that the hospital claimed the highest examination records, particularly in surgery, "in which subject it is approached by no other hospital." The trembling neophytes were also reminded that "another Lord Lister or Poet Laureate may be amongst you." The whirligig of time does, indeed, bring its revenges, for, though it is not of yesterday, some of us are old enough to remember the day when within the walls of this very institution Lister's introduction of antiseptic surgery was denounced as "a Scottish fad." Thanks largely to the "fad" the hospital in question has a great surgical record and the students have a right to be proud of it. Poetry in reference to the medical curriculum must, it is to be feared, be classed as a mere by-product.

